# Enhancing photocatalytic tetracycline degradation through the fabrication of high surface area CeO_2_ from a cerium–organic framework†

**DOI:** 10.1039/d4ra02640c

**Published:** 2024-05-30

**Authors:** Ayla Roberta Borges Serra, Gabriel Castro de Sousa, Viviane de Carvalho Gomes, Idio Alves de Sousa Filho, Cesar Koppe Grisolia, Baiwen Zhao, Richard I. Walton, Osvaldo Antonio Serra

**Affiliations:** a Department of Chemistry and Chemical Engineering, University Federal of São Carlos São Carlos Brazil; b Departament of Chemistry, FFCLRP, University of São Paulo Ribeirão Preto Brazil osaserra@usp.br; c Institute of Chemistry, Rural Federal University of Rio de Janeiro-UFRRJ Seropédica Brazil; d Department of Genetics and Morphology, Institute of Biological Sciences, University Brasilia-UnB Brasilia Brazil; e Department of Chemistry, University of Warwick Coventry CV4 7AL UK

## Abstract

Water pollution is a global environmental issue, and the presence of pharmaceutical compounds, such as tetracyclines (TCs), in aquatic ecosystems has raised growing concerns due to the potential risks to both the environment and human health. A high surface area CeO_2_ was prepared *via* atmospheric thermal treatment of a metal–organic framework of cerium and benzene-1,3,5-tricarboxylate. The effects of calcination temperature on the morphology, structure, light absorption properties and tetracycline removal efficiency were studied. The best activity of the photocatalysts could be achieved when the heat treatment temperature is 300 °C, which enhances the photocatalytic degradation performance towards tetracycline under visible light. The resulting CeO_2_ particles have high capacity for adsorbing TCs from aqueous solution: 90 mg g^−1^ for 60 mg L^−1^ TCs. As a result, 98% of the initial TC can be removed under simulated sunlight irradiation. The cooperation of moderate defect concentration and disordered structure showed tetracycline removal activity about 10 times higher than the initial Ce-MOF. An embryotoxicity assessment on zebrafish revealed that treatment with CeO_2_ particles significantly decreased the toxicity of TC solutions.

## Abstract 2

Water pollution is a global environmental issue, and the presence of pharmaceutical compounds, such as tetracyclines (TCs), in aquatic ecosystems has raised growing concerns due to the potential risks to both the environment and human health.

## Graphical abstract

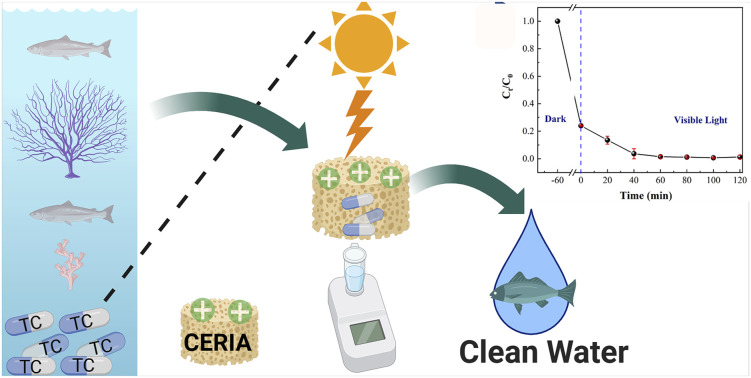

## 1. Introduction

Tetracycline antibiotics are a class of substances discovered in the 1940s and improved from natural to synthetic products since then. Tetracycline (TC) itself is one of the most important substances from natural sources of tetracyclines, also known as the first generation of the series.^1^ It is commonly used in both human and veterinary medicine to treat a variety of bacterial infections. While tetracyclines have been effective in combating bacterial infections, their use has raised concerns due to their potential impact on human health and the environment. The disposal of unused or expired tetracycline medications and excretion by humans and animals can result in the presence of tetracycline in water sources. This contamination can contribute to the emergence of antibiotic-resistant bacteria in aquatic environments. There is evidence that animal agriculture employs around 2300 tons of tetracycline antibiotics every year in Europe, which is about 66% of global antibiotic use.^2^ According to Xu *et al.* (2021),^3^ tetracycline antibiotics rank third in terms of regular use in Brazil, preceded by quinolones and penicillin.^3,4^ Europe uses around 2500 tons or more of tetracycline each year for the therapy of animals.^5^ Hence, developing efficient technologies to remove these organic pollutants from contaminated water is urgent.

To date, various methods have been introduced to remove contamination by organic compounds, including filtration,^6^ adsorption,^7^ and photocatalysis.^8^ Notably, the combination of adsorption and photocatalytic methods has proven a potential strategy to remove pollutants from wastewater without leading to secondary pollution.^9^ Adsorption–photocatalysis technology is recognized to be the most promising waste treatment strategy. The advantage is that adsorption and oxidation of organic compounds occur simultaneously, removing the necessity for two distinct processes.

Many different compounds have been tested in recent years for their ability to remove TCs from water.^10–15^ CeO_2_ has been one of the most studied lanthanide oxides in catalysis due its enhanced redox properties^16–19^ however, many advancements in CeO_2_ photocatalysts were generated by post-synthesis methods, which included the introduction of dopants, which makes processing complex. In addition, the majority of reports on the production of CeO_2_ using high-temperature calcination resulted in low specific surface area, limiting catalyst performance. In order to improve the photocatalytic TC degradation efficiency and stability of performance, it is important to optimize and create defective CeO_2_ photocatalysts which is realized by having poorly crystalline structures with surface active sites.

Metal–organic frameworks (MOFs) have been explored as templates to obtain oxides,^20,21^ leveraging their unique properties and structures with highly ordered and tunable porosity. Using MOFs as templates allows for the controlled synthesis of oxide materials with specific pore sizes and surface areas and this uniformity is crucial for achieving consistent and predictable properties in the resulting oxide, impacting its performance in various applications. This is particularly important for applications such as (photo)catalysis and adsorption. MOFs can be easily decomposed through thermal treatment, leaving behind the desired oxide material. This process allows for the production of high-surface-area oxides without the need for complex and harsh template removal procedures.^20^

In general, the few previous studies using cerium oxide for adsorption and photocatalysis of tetracycline under visible light radiation show low activity as a photocatalyst and therefore doping (metal or non-metal) is normally used to make it more efficient. Recently, He *et al.* studied CeO_2_ loaded soybean powder carbon as a photocatalyst for TC degradation.^21^ As a comparison, they tested CeO_2_ obtained by the cerium(iii) nitrate hexahydrate (CeH_3_NO_4_) treated with alkali and calcined at 600 °C under nitrogen without soybean powder carbon. The result, after 180 min indicate only 20% of TC removal. In another example, Su *et al.* showed that ceria synthesized under low temperature, with NaHCO_3_, photocatalysed only 20% of TC after 70 min of irradiation without significant adsorption.^22^ When the same material was doped with F, which gave a redshift of the adsorption, the conversion increased to 80%. Based on that, it is evident that the adsorption properties of CeO_2_ particles are influenced by the preparation procedure. Therefore, the preparation of porous CeO_2_ particles from decomposition of a cerium metal–organic framework (Ce-MOF) at low calcination temperature (300 °C) is investigated in this paper. The CeO_2_ particles are tested as adsorbent and photocatalyst for removing TCs from water. Powder X-ray diffraction (PXRD), scanning electron microscopy (SEM), nitrogen adsorption, and Fourier transform infra-red (FTIR) spectroscopy were used to characterize the CeO_2_ particles. In order to study TC adsorption onto the CeO_2_ particles, the key kinetic parameters and adsorption mechanisms have been investigated using adsorption models. Finally, to investigate the toxicity of the solution from which TCs were removed by CeO_2_ particles, a zebrafish embryo toxicity test has been applied.^23^

## 2. Materials and methods

### 2.1. Chemicals

All materials utilized in this study were of analytical grade. The materials used for synthesizing the Ce-MOF were cerium nitrate hexahydrate (Ce(NO_3_)_3_·6H_2_O, Fluka 99%) as the cerium precursor, benzene-1,3,5-tricarboxylic acid (H_3_BTC, Aldrich 98%) as ligand precursor and ethanol (Merck, CH_3_CH_2_OH), as solvent.

### 2.2. Ce–BTC synthesis

Ce–BTC was synthesized based on the method of Xiong *et al.* (2015),^23^ using a low-temperature solvothermal technique, with slight modifications to improve the final yield. 1.0 mmol of Ce(NO_3_)_3_·6H_2_O was dissolved in 2.0 mL of ultrapure water (solution A), and 1.0 mmol of H_3_BTC was dissolved in 18.0 mL of a mixture of water and ethanol with a volume to volume ratio of 1 : 1 (solution B). Then, drop by drop, solution A was added to solution B while the mixture was stirred with a magnetic stirrer and held in a water bath at 50 °C. The precipitate was collected from the reaction mixture after 30 minutes by centrifugation, washed multiple times with ethanol and ultrapure water, and dried at 70 °C for 24 hours. The MOF yield was 60% based on Ce(NO_3_)_3_·6H_2_O.

To confirm MOF formation and phase purity, PXRD, FTIR spectroscopy, and thermogravimetric analysis (TGA) investigations were carried out. The experimental characterization can be found in the ESI.† The FTIR spectrum in Fig. S1† confirmed the presence of carboxylate bridged species. TGA, Fig. S2,† confirmed the expected chemical formula for Ce-MOF as Ce(BTC)·6H_2_O. Water was removed in one step at 120 °C (calc. 23.73%, found 23.5%). Thermal decomposition culminated in an event at 350 °C, which was associated with decomposition of the benzene tricarboxylate anion; the CeO_2_ residue was formed at 800 °C (calc. 37.98%, found 37.88%).

### 2.3. CeO_2_ preparation

The CeO_2_ particles were prepared by Ce-MOF thermal decomposition.^24^ The as-obtained Ce-MOF was calcined in an air atmosphere at 300, 500, 700, or 900 °C for 2 h at a heating rate of 10 °C min^−1^.

### 2.4. Characterization of the materials

Powder X-ray diffraction (D5005 diffractometer, Siemens, Germany) was performed with Cu-Kα_1/2_ radiation (1.5418 Å) scanned at 2° min^−1^. For the prepared CeO_2_ particles, the crystallite sizes and lattice parameters were determined from the measured PXRD patterns. The lattice parameters were determined by Rietveld refinement using MAUD software using COD 9009008 as standard cubic CeO_2_, and the Scherrer equation was used to determine the average crystallite size to the most intense peak.

A NOVA 4200e Quantachrome (USA) system was used to evaluate the N_2_ adsorption–desorption isotherms at 77 K. Before the adsorption isotherm was acquired, the CeO_2_ particles (0.15–0.17 g) were degassed at 150 °C for 3 h. The Brunauer–Emmett–Teller (BET) method was used to calculate their specific surface area. After applying the Barret–Joyner–Halenda (BJH) method to the desorption isotherms, the pore size distribution was calculated. The overall pore volume was calculated using the ratio *p*/*p*_0_ = 0.99.

The thermogravimetric analyses were performed on a Q600 (TA instruments) analyzer. Samples were heated at a rate of 10 °C min^−1^, from room temperature to 1000 °C, with simultaneous measurement of TGA-DTA-DSC in a synthetic air atmosphere. The thermogravimetric (TG) and differential scanning calorimetry (DSC) curves were acquired using aluminum oxide (Al_2_O_3_) as an inert reference material.

A TPR/TPD Micromeritics AutoChem 2920 system that was fitted with a thermal conductivity detector, was used for H_2_ temperature-programmed reduction (H_2_-TPR) investigations. For the H_2_-TPR experiments, the samples were pretreated up to 300 °C (10 °C min^−1^) in air (50 mL min^−1^) for 1 h, followed by an increase in temperature to 900 °C (10 °C min^−1^) by employing 10% H_2_/air flow (50 mL min^−1^). Temperature-programmed desorption (TPD) was evaluated in a micro-reactor system coupled with a Pfeiffer Omni Star mass spectrometer, and the fragments *m*/*z* = 2 (H_2_), 44 (CO_2_), and 78 (benzene) were observed.

Zeta potential measurements were performed using the ZetaSizer Nano ZS (Malvern) system, model ZEN 3601 with the software Software PSS0012 Malvern Instruments DTS Application to collect the data.

## 3. Results and discussion

### 3.1. Properties of CeO_2_ particles

**Fig. 1:**
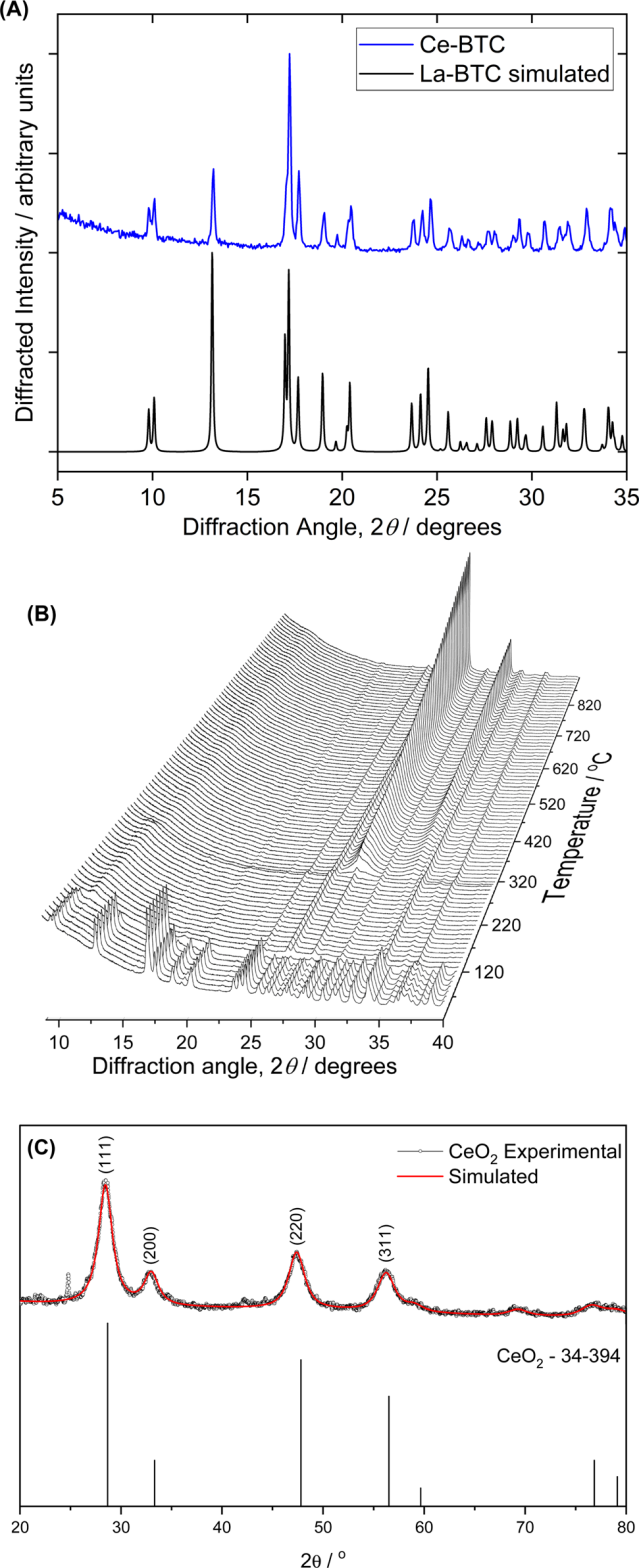
(A) Powder X-ray diffraction pattern of Ce-BTC compared to the simulated pattern of La(BTC)·6H_2_O (CCDC 290771) (B) PXRD powder patterns of Ce-MOF recorded *in situ* during heating. Note that peaks that persist throughout the measurement are due to the sample holder. (C) Rietveld refinement (Maud software) of CeO_2_ particles. Experimental (black line) and simulated (red line).

Fig. 1A shows the PXRD pattern of the Ce-MOF, which has crystalline structure as previously reported in the literature for the lanthanum analogue. The pattern of the compound matches that of La(BTC)·6H_2_O (CCDC 290771). The peak positions and their Miller indices are shown in Table S1.† X-ray powder thermo-diffractometry was conducted from room temperature to 900 °C to investigate how the Ce-MOF behaved during conversion to CeO_2_ (Fig. 1B). The MOF structure persisted from room temperature to about 100 °C, when an amorphous material corresponding to water loss (observed by TGA, Fig. S2†) emerged between 100 to 300 °C according to Fig. 1B. CeO_2_ crystallization started at 300 °C, in agreement with the ligand combustion observed during the Ce-MOF TGA analysis. The PXRD of the CeO_2_ obtained after the calcination at 300 °C of the Ce-MOF is shown in Fig. 1C. The materials display broad PXRD peaks, indicating their low crystallinity, but the patterns agree with the typical PXRD pattern of the face centered fluorite structure of CeO_2_ (JCPDS PDF #34-0394).

**Fig. 2:**
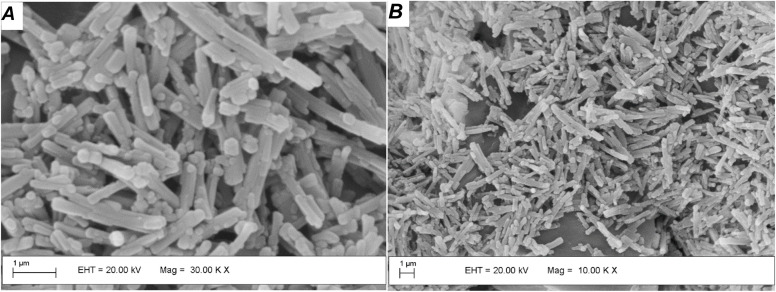
SEM images of (A) Ce-BTC MOF and (B) derived CeO_2_ particles obtained at 300 °C.

SEM micrographs were acquired to evaluate the morphological characteristics of the CeO_2_ particles. Fig. 2 shows the morphologies of Ce-MOF and the derived CeO_2_ particles. A large amount of nanorod particles after Ce-MOF calcination at 300 °C was detected, reflecting the morphology of the starting material. This phenomenon has been reported by other synthetic approaches to ceria from different precursors.^25^ SEM micrographs for the oxides obtained at higher temperature sintering becomes noticeable and rises with calcination temperature over 500 °C, at which point a combination of nanorods and spherical morphologies is seen (Fig. S3†).

**Fig. 3:**
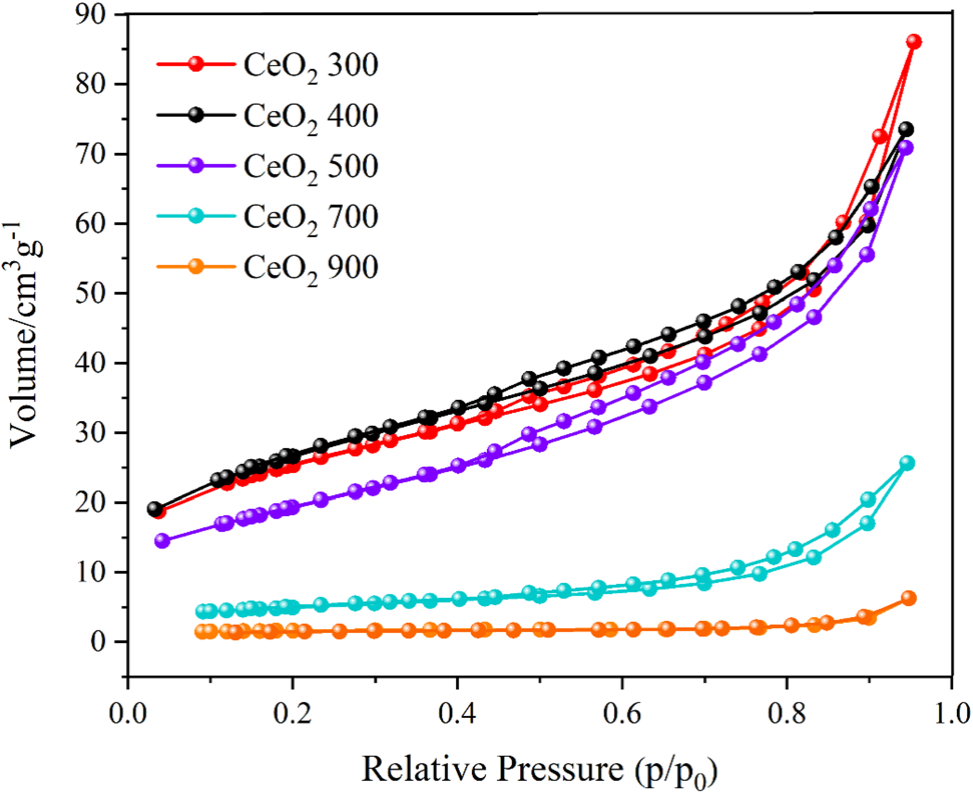
Isotherms obtained from the analyses of nitrogen physisorption. The calcination temperature (°C) used in the sample preparation is indicated in the legend.

To evaluate the surface area and pore size of the CeO_2_ materials, Brunauer–Emmett–Teller (BET) analysis of nitrogen adsorption isotherms was performed (Fig. 3). Samples prepared by different calcination temperatures between 300 and 900 °C were studied to determine the effect of temperature on the resulting particles. The CeO_2_ obtained at lower calcination temperature, 300 °C, had larger surface area than that obtained at 500 °C: 95.05 and 69.01 cm^3^ g^−1^, respectively. The surface area of the particles formed at lower calcination temperatures exceeded the predicted range, indicating that they preserved the Ce-MOF characteristics. The materials' adsorption capacity slightly increased as the relative pressure *p*/*p*_0_ varied between 0 and 0.6, pointing to the presence of micropores. Rapid uptake was observed for *p*/*p*_0_ ranging from 0.8 to 1.0, indicating mesoporous structures. The adsorption isotherm exhibited a hysteresis loop, indicating that the materials had both microporosity and mesoporosity_._^26,27^ Given its higher surface area, the CeO_2_ prepared by calcination at 300 °C was selected for further studies of its adsorption and catalysis, as detailed in the following sections.

**Fig. 4:**
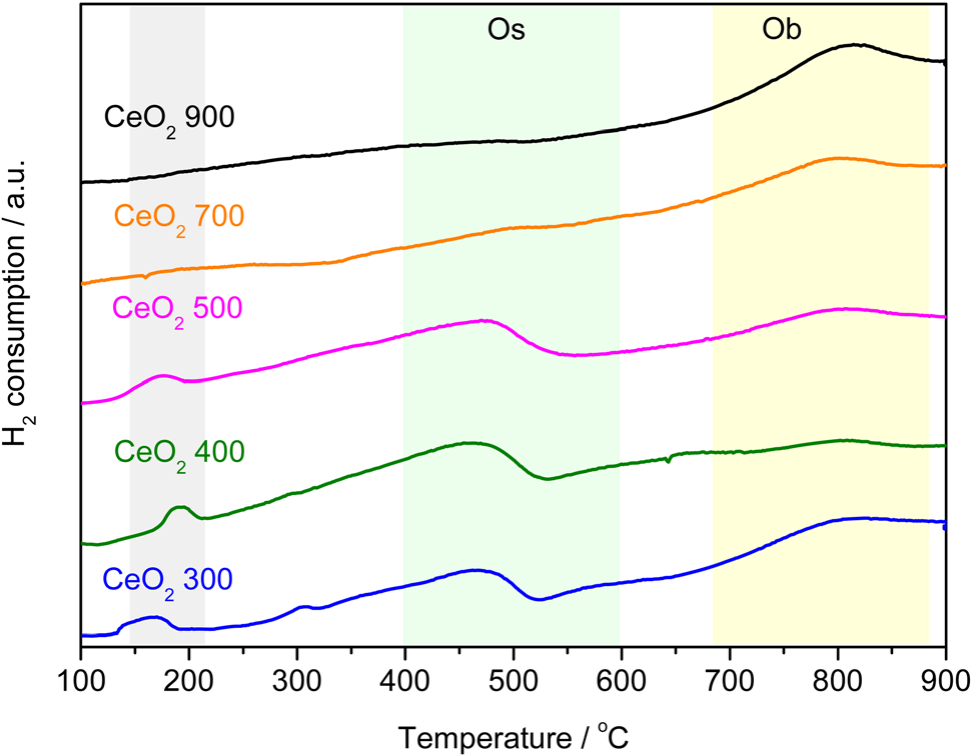
H_2_-TPR profiles of CeO_2_ obtained at different calcination temperatures.

H_2_-TPR was employed to investigate the effect of thermal decomposition of the Ce-MOF on the reducibility of the CeO_2_ catalysts. TPR profiles for CeO_2_ samples are depicted in Fig. 4. TPR profiles agree with those reported in the literature, with two main features detected due to the reduction of ceria by H_2_: one between 300 and 600 °C and the other between 650 and 900 °C.^28–30^ The peaks are related to the removal of surface (O_s_) and bulk oxygen (O_b_) ions, respectively. Table 1 summarizes the behaviour of H_2_ consumption by cerium oxides evaluated in this work. The samples calcined at 700 and 900 °C do not have reducible species on the surface, in agreement with the results described by Zhang *et al.* (2020) who synthesized and calcined CeO_2_ at different temperatures and showed H_2_ consumption similar values to those here reported.^30^ They confirmed that increasing the calcination temperature reduces the surface area and increases crystallinity, resulting in less reducible oxygen species on the material's surface. On the other hand, samples obtained by calcination at lower temperatures showed a higher H_2_ consumption. According to Fig. 4, the same was observed in this work. Furthermore, these materials showed a peak at low temperature in the H_2_-TPD curves (150–200 °C), the peak also refers to the consumption of H_2_, since it was also observed in the mass spectrometer (*m*/*z* = 2), Fig. S4.† Reductions in this region are usually observed for noble metals or even for CeO_2_ doped with noble or transition metals.^31,32^ Organic residues (as carbon) from the decomposition of the original MOF could be retained on the surface of CeO_2_, promoting some type of interaction that favors the consumption of H_2_ and catalyst surface reduction. The TPR analysis indicates that the determining factor in the reduction of ceria is the loss of surface area due to the calcination temperature, a process that is increased by the textural and structural evolution and that promotes sintering, because of the specific area. The reducibility of ceria prepared using MOF as a precursor is significantly higher than that observed in CeO_2_ prepared by other techniques, for example as compared to materials reported by Zhang *et al.*^33^

**Table 1:** Total consumption of H_2_ (mmol g^−1^)

Peak region	Sample			
	CeO_2_-300	CeO_2_-500	CeO_2_-700	CeO_2_-900
O_s_ peak	0.42	0.62	0.07	0.07
O_b_ peak	0.60	0.36	0.57	0.79

### 3.2. TC adsorption onto CeO_2_ particles

**Fig. 5:**
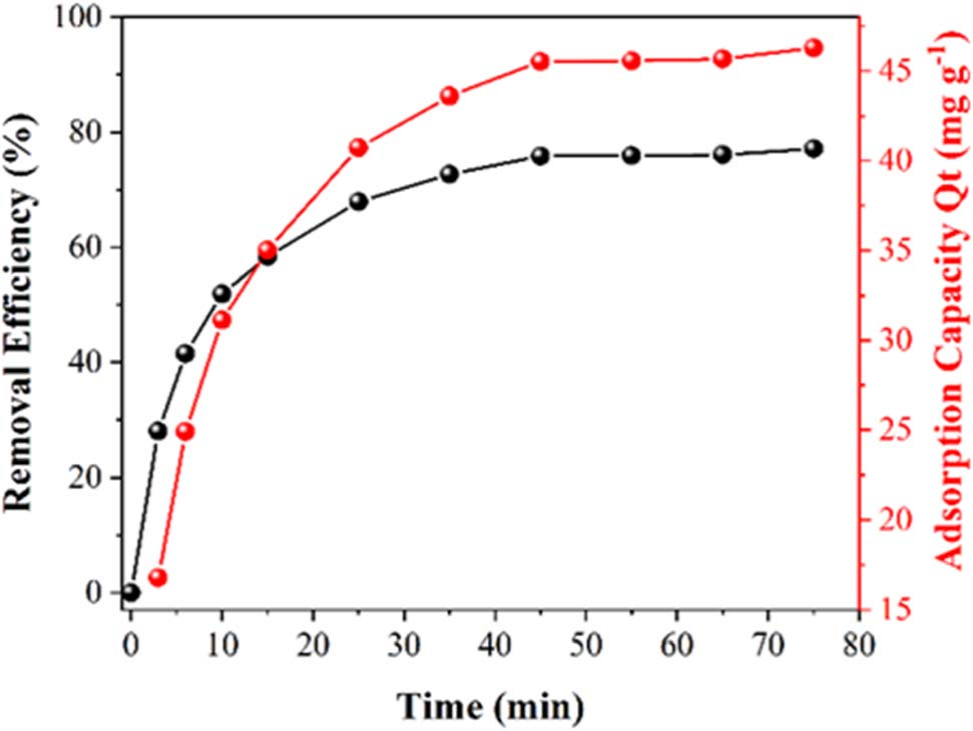
The efficiency of TC removal and the adsorption capacity (*Q*_*t*_) of CeO_2_ as a function of contact time.

In the ESI,† adsorption and photocatalytic studies are discussed in detail. Fig. S5† shows the UV-Vis spectral monitoring of TC adsorption onto CeO_2_ particles from aqueous solution *vs.* contact time for an initial concentration of 30 mg L^−1^ at room temperature. The effectiveness and adsorption capacity in removing TC as a function of contact time are plotted in Fig. 5.

**Fig. 6:**
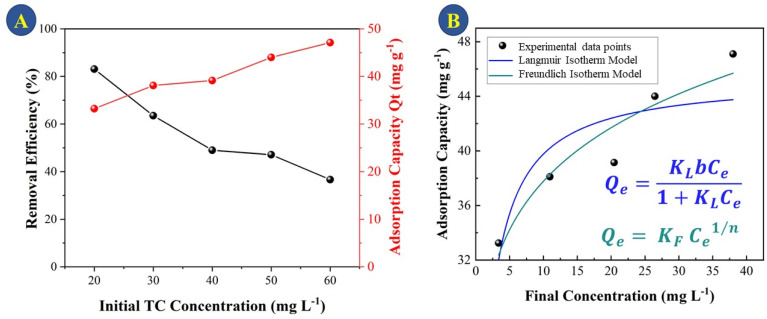
(A) TC removal efficiency and adsorptive capacity (*Q*_*t*_) of CeO_2_ as a function of initial TC concentration (B) adsorption isotherms of TC on CeO_2_.

It can be observed that during the first 15 min there is a high rate of adsorption of TC on the surface onto CeO_2_ and equilibrium is reached after 50 min of contact. At the end of the treatment, there is a total adsorption of 76% TC. The high adsorption rate and the fact that equilibrium is reached quickly indicated that the CeO_2_ is an efficient TC adsorbent. Large surface area and high porosity can furnish materials with high adsorption capacity, but reaching the adsorption equilibrium can be slow, as in the case of activated carbon.^34^ Indeed, a previous work reported that TC adsorption onto activated carbon took 8 h to reach equilibrium,^35^ which was much slower compared to the CeO_2_ studied here. Similar results were obtained when TC was adsorbed onto porous carbon (equilibrium took 8 hours when the TC concentration was between 25 and 40 mg L^−1^)^36^ and on graphene oxide (90 min).^37^ An adsorption capacity at equilibrium (*Q*_e_) of 46.3 was obtained for the CeO_2_ studied here. Fig. 6 depicts the efficiency of TC removal and the adsorption capacity of CeO_2_ in relation to varied initial TC concentrations at 303 K and a contact period of 60 min. As shown in Fig. 6A, the adsorption capacity increases as the TC concentration increases, up to a maximum value of 60 mg L^−1^ TCs. The rapid TC adsorption during the first 30 minutes suggests that the CeO_2_ surface has a significant number of adsorption sites. After 30 min, it is observed TC adsorption slightly increases up to 60 min. Therefore, this time was used for further studies, to reach adsorption equilibrium. After 60 min, TC adsorption was not significant, which may be due to repulsive interaction of adsorbed molecules. For comparison, the TC adsorption capacity of CeO_2_ obtained by precipitation was reported as 18 mg g^−1^ at an initial TC concentration of 25 mg L^−1^ and 38 mg g^−1^ at 125 mg L^−1^ TCs.^38^ Thus, the adsorptive removal performance of the CeO_2_ prepared by Ce-MOF decomposition was higher compared to other CeO_2_ adsorbents reported in the literature. A comparative adsorption test was conducted on the Ce-MOF to evaluate the efficacy in relation to the obtained oxides. The results, shown in Fig. S6,† indicate a very low activity of approximately 10% removal following the adsorption and photocatalytic method. The results indicate the relevance of carefully controlling the process of calcination of the MOF, in order to achieve superior oxides.

Using two isotherm models, Langmuir and Freundlich, the experimental data for TC adsorption onto CeO_2_ particles at room temperature were fitted.^11^ The Langmuir isotherm equation is as follows eqn (1).1
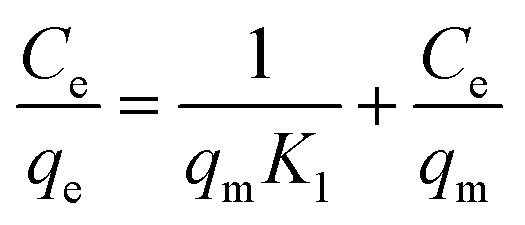


The equation describes the formation of a uniform monolayer of molecules after adsorption at a surface, with no interaction between adsorbed molecules. *K*_1_ is the Langmuir constant, which represents the strength of the adsorption capacity, and *C*_e_ is the equilibrium concentration of TC in the solution (expressed in mg L^−1^), *q*_e_ is the quantity of TC adsorbed at the equilibrium concentration, and *q*_m_ is the maximum amount of TC adsorbed.^39^

The Freundlich model is used when the adsorption occurs in a heterogeneous way through the formation of multilayers, with an interaction between the adsorbed molecules.^15^ This model suggests that adsorption occurs at all active sites on the surface, in addition, there may be different types of adsorption sites with different energies. Typically, the equation eqn (2) is represented in logarithmic form:2
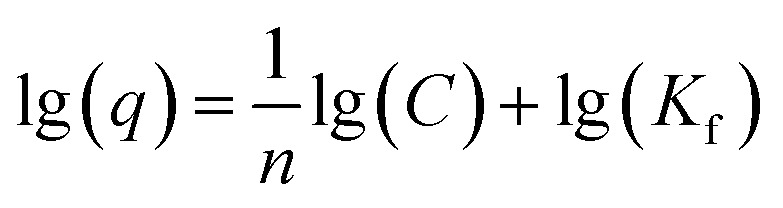
where 1/*n* denotes the degree of difficulty of adsorption and *K*_f_ denotes the Freundlich adsorption constant, which represents adsorption ability. According to previous work, when the value of 1/*n* is less than 1, the adsorption process can be interpreted as favorable and spontaneous.^40^ Using the Langmuir and Freundlich models, linear fits were performed on the TC adsorption data on CeO_2_, and the results are displayed in Fig. 6B. The best fit for TC adsorption was using the Freundlich model, since it presented the best correlation coefficient (*R*^2^ = 0.917), indicating that adsorption occurs in multilayers and presents adsorption sites with different energies.

### 3.3. TC adsorption kinetics

**Fig. 7:**
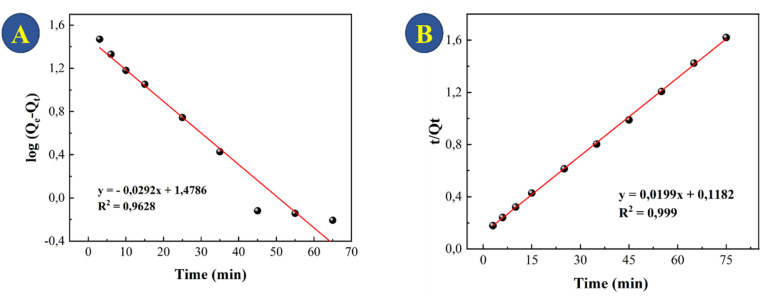
Models of the kinetics of TC adsorption onto CeO_2_ at a concentration of 30 mg L^−1^: (A) pseudo-first order and (B) pseudo-second order.

The adsorption data were used to test pseudo-first-order (Fig. 7A) and pseudo-second-order (Fig. 7B) models. The choice of the best model accounted for the adsorption kinetics was based on the correlation coefficient (*R*^2^), with the highest *R*^2^ value provided by the pseudo-second order kinetics (Table 1). This model explains that adsorption involves processes, most notably chemisorption and physisorption. This includes covalent bonding and ion exchange, which include electronic interactions between the adsorbent and adsorbate.^38,40–42^

Thermodynamic parameters were determined to understand the nature of the adsorption process using the experimental data of TC adsorption onto CeO_2_ at different temperatures (Fig. S7†).^43^ This allows determination of whether the adsorption process is physical or chemical, whether it is endothermic or exothermic, and whether it is spontaneous or non-spontaneous. Thermodynamic tests were performed by using 50 mg L^−1^ TCs. This concentration was chosen because it was high enough not to adsorb all the molecules after the contact time, allowing the adsorbed concentration determination. The plotted ln *K*_d_*vs.* 1/*T* (Fig. S7B†) gave a linear relationship that was used to calculate the thermodynamic parameters. Table 2 lists the changes in Gibbs free energy change (Δ*G*), enthalpy (Δ*H*), and entropy (Δ*S*) obtained by using eqn (3) and (4):^44^

3 Δ*G* = −*RT* ln *k*_d_

4
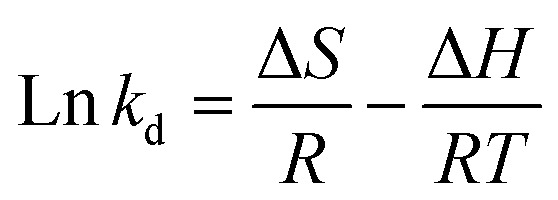
where *R* and *T* represent the gas constant (8.314 J mol^−1^ K^−1^) and the absolute temperature (K), respectively, *K*_d_ represents the equilibrium constant, and *Q*_m_ (mL mg^−1^) represents the maximum adsorption capacity. From the slope and intercept of the graph of ln *K*_d_*vs.* 1/*T*, the values for Δ*H* and Δ*S* (Fig. S7B†) were determined. The Δ*G* values were calculated at 343, 333, 323, 313, and 303 K using eqn (3).

**Table 2:** Thermodynamic parameters for TC adsorption onto CeO_2_

*K*_d_ (mL mg^−1^)	0.02	0.049	0.706	1.611	1.611	Δ*H* (kJ mol^−1^)	Δ*S* (J mol^−1^ K^−1^)
Temperature (K)	343	333	323	313	303	−12.676	−40.813
*Q*_e_ (mg g^−1^)	0.5	1.2	13.04	22.3	22.3		
Δ*G* (kJ mol^−1^)	−102.9	−106.3	−109.7	−113.1	−116.5		

TC adsorption on the surface of the CeO_2_ materials is a spontaneous process due to negative values of Δ*G* for the temperatures studied. There is an increase in Δ*G* values with increasing temperature and this behaviour indicates that the adsorption is energetically more spontaneous at lower temperatures.^45^ The enthalpy of adsorption of TC is an exothermic process, which differs from previous reports of the adsorption of minocycline onto CeO_2_.^46^ Therefore, the adsorption behaviour on the surface of CeO_2_ will be directly related to the type of molecule adsorbed. The negative Δ*S* value confirmed that randomness at the liquid–solid interface was reduced as well as the affinity of the adsorbent for adsorption.

The magnitude of Δ*H* can be used to achieve a more detailed view of the nature of the adsorption binding energy and it is possible to predict that the amine and ketone groups on TC are bound to the adsorption sites of the CeO_2_, because the energies associated with hydrogen bonding range from 5 to 20 kJ mol^−1^.^40^

### 3.4. Photocatalytic study

**Fig. 8:**
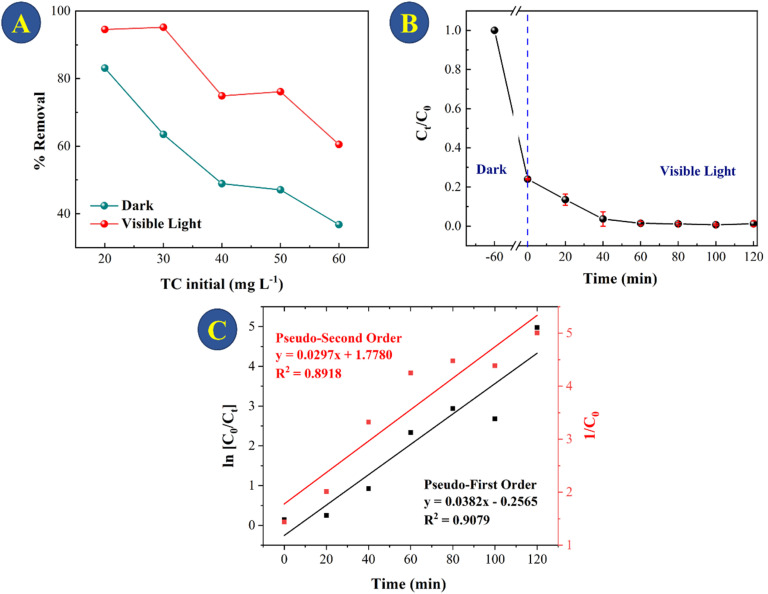
(A) TC removal in the dark (60 min) and under visible light (120 min) for different TC concentrations; (B) TC degradation performance of the photocatalyst (the CeO_2_ particles) under simulative sunlight irradiation for 30 mg L^−1^ TCs; (C) black colour: fitting of the reaction kinetics as pseudo-first order (ln[*C*_0_/*C*_*t*_] *vs. t*), kinetic constant *k* = 0.0382 min^−1^; red colour: fitting of the reaction kinetics as pseudo-second order (1/[*C*_0_] *vs. t*), kinetic constant *k* = 0.0297 mg^−1^ min^−1^.

For treatment of real systems, the initial concentration of the pollutant is important to consider for a credible removal to be achieved. Fig. 8A shows the adsorption and photocatalytic activity of the CeO_2_ for 20–60 mg L^−1^ TC degradation. As expected, the initial TC concentration plays a substantial role in the adsorption process: at initial TC concentrations of 20, 30, 40, 50, and 60 mg L^−1^, 81%, 60%, 42%, 40%, and 36%, respectively, of TC was removed. The fact that the CeO_2_ was able to remove the majority of the TCs within 60 minutes of adsorption despite the relatively low initial TC concentration is of critical significance for practical application. The CeO_2_ particles showed relatively high photocatalytic activity toward TC degradation within 2 hours of being exposed to light. When TC concentration was increased from 20 to 30 mg L^−1^, the ability of CeO_2_ to catalyze photodegradation decreased by just 5%. However, when the TC concentration was 50 mg L^−1^, catalytic TC photodegradation decreased by 42%. This is likely to be because at greater TC concentrations, decomposition products of TC compete with photocatalytic and adsorption sites at the surface of the CeO_2_.^47^ Considering the results, the best photocatalytic degradation test was using 30 mg L^−1^ TCs, so this concentration was used to evaluate the kinetics of photocatalytic TC degradation (Fig. 8B and C). The photocatalytic TC degradation was determined as having pseudo-first-order kinetics by linear regression analysis (*R*^2^ = 0.9078, Fig. 7C).

When the data collected in this study for tetracycline removal are compared to those obtained using commercial CeO_2_, a significant difference in the adsorption and photocatalysis processes is found. For 30 mg L^−1^ of TC, the removal using the commercial CeO_2_ was near 10% after 2 h of photocatalysis, as shown in Fig. S8.† Table S2† shows the results for the oxides, CeO_2_-300, CeO_2_-500, CeO_2_-700 and CeO_2_-commercial for comparison between adsorption and photocatalysis for TC solution of 30 mg L^−1^.

In order to achieve a deeper comprehension of the adsorption–photocatalysis properties of this material, we conducted zeta potential studies at a pH of 6 for all oxides, from 300 to 700 °C, as well as for commercial CeO_2_. Based on the results, showed in Table S3,† the adsorption behaviour can be explained mostly by the surface charge of the oxide obtained. The CeO_2_-300 exhibit a very high potential of +33 mV, which became more negative according to the increase of the calcination temperature. For CeO_2_-900, for example, the potential is −5.3 mV and −10 mV for the TC solution in the same pH (6). Considering these data and knowing that a positive zeta potential, as in the case of CeO_2_-300, is a reflection of its positive residual charge on the surface of the material, which tends to attract a negative charge such as TC, we are able to explain the adsorption process as an electrostatic effect.

Therefore, after TC adsorption, when the excitation source is turned on, the TC adsorbed on CeO_2_ begins to be degraded. As the system proceeds, the adsorbed TC is degraded, allowing the remaining TC in the solution to be adsorbed again. As this is not a case of photocatalysis alone, there is always concomitant adsorption, the regeneration of CeO_2_ is not rapid. It is necessary to keep the system under excitation for 60 min so that CeO_2_ can be reused with 50% efficiency (Fig. S9†). Following a 60 minutes regeneration time, the XRD pattern indicates that the structure remains unaltered after the adsorption and photocatalysis test, as indicated in Fig. S10.†

CeO_2_-300, in addition to having the highest positive surface charge, also has the smallest band-gap of 2.5 eV. For CeO_2_ obtained at 700 °C, the band-gap are 2.55 and for commercial CeO_2_ 2.88 eV (Fig. S11†). A smaller band gap means that CeO_2_ can absorb photons with lower energy, including those in the visible light region. This broadens the range of light that can be utilized for photocatalytic reactions, enhancing the efficiency of the photocatalyst under visible light irradiation. Oxygen vacancies or defects can be related to the smaller band-gap for CeO_2_-300. Poorly crystalline oxides often have more defects and vacancies compared to highly crystalline ones. This is because their disordered structures inherently contains more vacancies and defects. In highly crystalline materials, defects are minimized, although they can still occur at low levels due to imperfections in the crystal lattice or during the fabrication process. The higher concentration of defects and vacancies in poorly crystalline oxides can influence their properties such as electrical conductivity, optical behaviour, and chemical reactivity.

### 3.5. Toxicity study

**Fig. 9:**
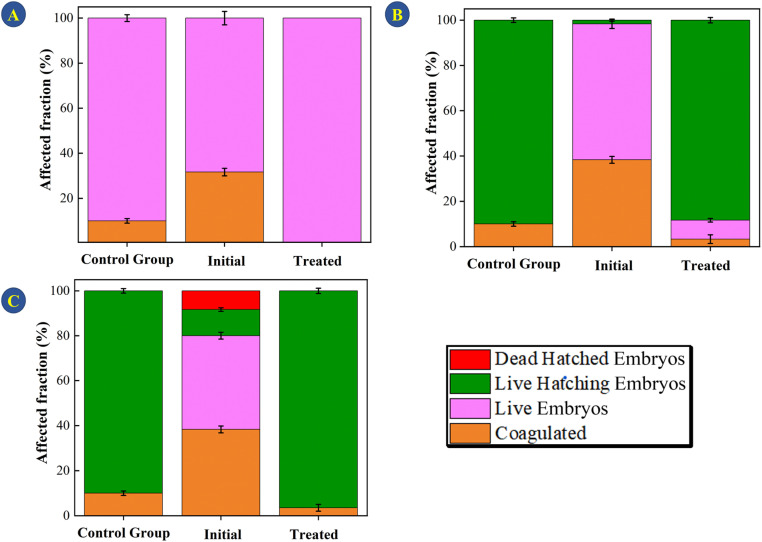
Mortality of zebrafish embryos exposed for (A) 24, (B) 72, and (C) 144 hours to the control group, the initial TC solution (30 mg L^−1^), and the TC solution treated with the CeO_2_ and irradiated with light.

The methodology for evaluating toxicity is given in the ESI.† Evaluating toxicity is very important when it comes to adsorption and photocatalytic processes given that, in some cases, the degradation products can be even more toxic than the starting material.^48^ For this reason, the TC solution (30 mg L^−1^) was evaluated before and after submitting it to photodegradation catalyzed by the CeO_2_ particles (Fig. 9). Embryological development monitoring of zebrafish is possible due to their transparent chorion (the outer membrane that protects the embryos), in addition, such species are extremely sensitive to chemical variations in the environment where they are, especially in early stages of development.^49^ Thus, fish embryo acute toxicity (FET) is ideal and commonly used as an indicator of ecotoxicity.^50,51^Fig. 9 shows the FET results for the assays conducted for 24, 72, and 144 h.

**Fig. 10:**
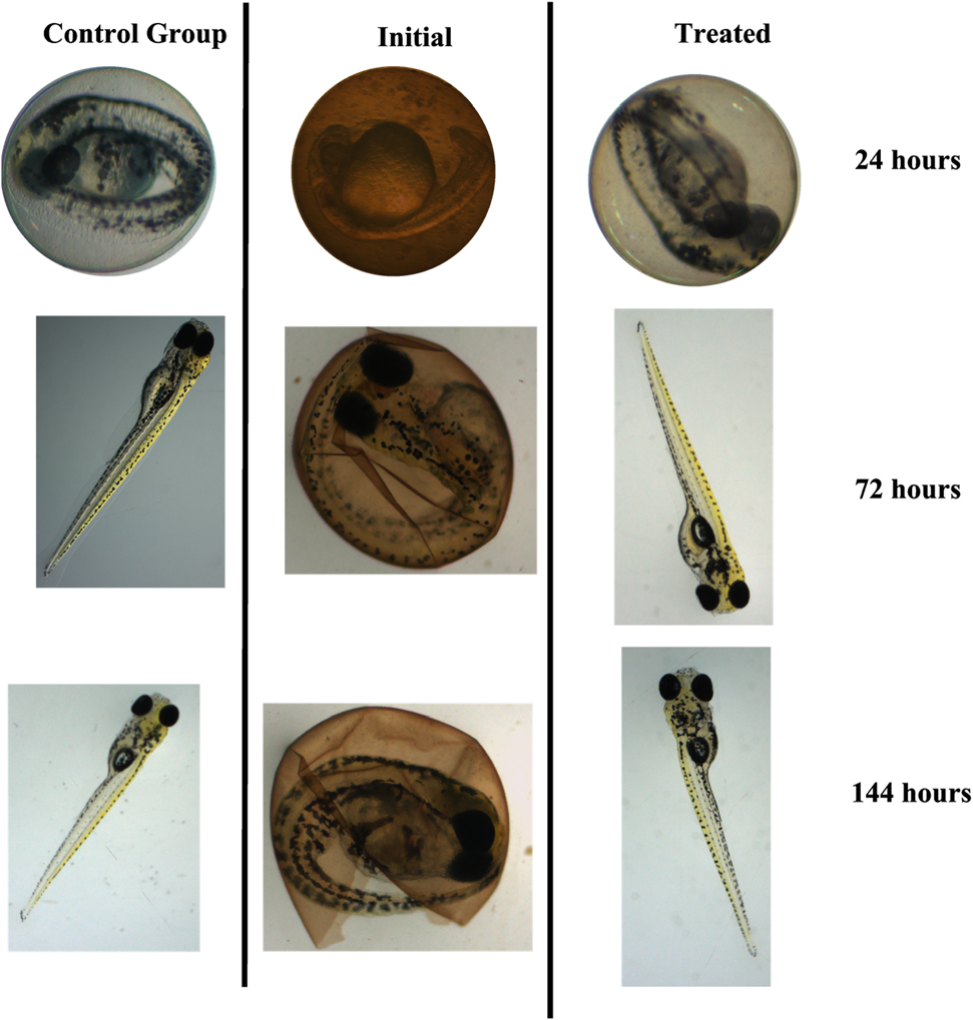
Observations of the development of zebrafish embryos and larvae made during FET at different times of exposure to the control, initial TC solution, and TC solution treated with the CeO_2_. (Stereomicroscope Stemi 2000, Zeiss, magnification of 25×).

The initial TC solution delayed hatching at all the evaluated times. After 144 h, the final hatch rate was lower as compared to the control and the TC solution treated with the CeO_2_. The treated solution did not differ significantly from the control group at the end of the 144 h assay, showing that the treatment eliminated the effects promoted by TCs (One Way ANOVA, Dunnet test, *p* < 0.05). In addition, no malformation was observed in the TC solution or in the treated solution (Fig. 10). In terms of mortality after 144 hours, embryos exposed to the treated solution did not vary statistically from the control group (One Way ANOVA, Dunnet test, *p* 0.05), demonstrating that the CeO_2_ effectively removed the organic load and harmful effects of the original TC solution.

## 4. Conclusions

High surface area CeO_2_ was obtained from decomposition of a Ce-MOF at low temperature. The CeO_2_ displayed significant TC adsorption performance compared to other oxide materials reported in the literature. The oxides synthesized at elevated temperatures (500 and 700 °C) exhibit reduced adsorption and low photocatalytic activity (ESI†). The kinetic experiments were conducted exclusively on the oxide synthesized at 300 °C where in the initial TC concentration range between 20 and 30 mg L^−1^, the CeO_2_ shows outstanding photocatalytic degradation performance. The temperature of calcination affects not only the surface area but also the surface charge of the oxide produced. Since TC adsorption proceeds through electrostatic contact, CeO_2_-300 demonstrated the highest activity. Poorly crystalline oxides frequently contain more defects and vacancies than highly crystalline ones, which may also explain why CeO_2_-300 exhibits the best photocatalytic activity efficiency. Freundlich and pseudo-second-order kinetic models described TC adsorption on CeO_2_. According to the result of the thermodynamic investigation, the adsorption of TC is an exothermic and spontaneous process, making it an environmentally benign process. The hypothesis that the adsorption of TC on zebrafish eggs can perturb microbial communities was tested and showed an excellent activity of CeO_2_ with no sign of adverse outcome on the FET assay. The current contribution highlights attention to the significance of CeO_2_ in perspective of its interaction with biological systems, revealing a competitive tool that can be applied to real-world situations. In order to apply these materials in the enhanced treatment of water polluted with antibiotics, we have acquired a thorough understanding of the TC elimination mechanisms in water. We also note that the high surface area CeO_2_ that is reported here may also have properties suitable for application in their heterogeneous catalysis applications. The optimal removal and low cost of CeO_2_ preparation are extremely advantageous for practical applications.

## Data availability

All data generated or analyzed during this study are included in this published article [and its ESI†].

## Author contributions

Material preparation, data collection and analysis were performed by Ayla Roberta Borges Serra, Gabriel Castro de Sousa, Viviane de Carvalho Gomes, Idio Alves de Sousa, and Baiwen Zhao. The FET test was evaluated by Cesar Koppe Grisolia and Idio Alves Sousa. Kinetic and thermodynamic studies were conducted by Idio Alves de Sousa and Ayla Roberta Borges Serra. The first draft of the manuscript was written by Ayla Roberta Borges Serra. The first manuscript draft of Ayla Roberta Borges Serra was evaluated by all writers. Osvaldo Antonio Serra and Richard I. Walton revised the text. All authors read and approved the final manuscript.

## Conflicts of interest

We declare no competing interests.

## Supplementary Material

RA-014-D4RA02640C-s001
