# Supplementary material for: Enhancing photocatalytic tetracycline degradation through the fabrication of high surface area CeO2 from a cerium–organic framework
Source: RSC Adv. 2024 May 30;14(25):17507–18. doi: 10.1039/d4ra02640c (PMC11138135; doi:10.1039/d4ra02640c)
Supplement: RA-014-D4RA02640C-s001 [file RA-014-D4RA02640C-s001.pdf]

Electronic Supplementary Material (ESI) for RSC Advances.
This journal is © The Royal Society of Chemistry 2024

Support information

Enhancing photocatalytic tetracycline degradation through the fabrication of high surface area CeO_2 from a cerium-organic framework.

Ayla Roberta Borges Serra[1], Gabriel Castro de Sousa[2], Viviane de Carvalho Gomes[2], Idio Alves de Sousa Filho[3], Cesar Koppe Grisolia[4], Baiwen Zhao[5], Richard I. Walton[5], Osvaldo Antonio Serra[2*]

1. *Department of Chemical Engineering, Federal University of São Carlos-UFSCar, São Carlos, SP, Brazil*
2. *LTR, Departamento de Química, FFCLRP, Universidade of São Paulo-USP, Ribeirão Preto, SP, Brazil*
3. *Institute of Chemistry, Rural Federal University of Rio de Janeiro-UFRRJ, Seropédica, RJ, Brazil*
4. *Department of Genetics and Morphology, Institute of Biological Sciences, University Brasilia -UnB, Brasilia, DF, Brazil*
5. *Department of Chemistry, University of Warwick, Coventry CV4 7AL, U.K.*

* corresponding author's email address: osaserra@usp.br

Adsorption experiments

The temperature used for the TC adsorption experiment was 25 °C. TC adsorption studies were carried out in a concentration range from 20 to 50 mg L^{-1}. Then, 12.5 mg of CeO_2 was added to 25 mL of TC solutions while being constantly stirred. Following that, 2 mL aliquots were collected every 20 minutes, centrifuged at 14.500 rpm for 10 minutes, and the supernatant was analyzed using UV-Vis. (**Fig S3**). Eq. 1 was used to determine the concentration of adsorbed TC:

$$Q_t = \frac{V(C_o - C_f)}{M} \quad (1)$$

where C_0 represents the initial TC concentration (mg L^{-1}), C_f represents the final TC concentration at time t (mg L^{-1}), Qt represents the adsorptive capacity, V represents the TC solution volume (L), and M denotes the adsorbent mass (g).

Pseudo-first order and pseudo-second order equations were used to examine the kinetics, Eqs. 2 and 3, respectively (El-Latif et al., 2013; Ho and McKay, 1999).

$$\log(Q_e - Q_t) = \log q_e - {k^t}/{2.303} \quad (2)$$

where k is the adsorption rate constant (min^{-1}) and Q_e and Q_t are the amounts of adsorbed TC (mg g^{-1}) at equilibrium and at specific times t (min). The slope of the line log (Q_e-Q_t) *vs* t can be used to determine the constant k.

$$\frac{t}{Q_t} = \frac{1}{kQ_e^2} + \frac{1}{Q_e}t \quad (3)$$

where k_2 is the pseudo-second-order constant (g mg^{-1} min^{-1}) and Q_e and Q_t are the amounts of adsorbed TC (mg g^{-1}) at equilibrium and at specified times t (min), respectively.

Furthermore, the effect of temperature was evaluated at different temperatures – 303, 313, 323, 333, and 343 K.

Photocatalytic experiment

The photocatalytic activity of the CeO_2 particles was assessed by TC photodegradation under simulative sunlight—a 300W Xenon lamp (λ ~ 280–420 nm) was applied after the TC adsorption process. The same range of TC concentration employed in the TC adsorption experiments was used in the photocatalytic degradation experiments. First, the solution containing the CeO_2 particles and TCs was agitated at 1000 rpm for 60 minutes in a dark environment to ensure that no further adsorption-desorption processes occurred. After that the system was located under visible light irradiation conditions for 2 h. Finally, the TC concentrations during adsorption and the photocatalytic degradation process were analyzed as described in section 2.5.

The Ce-MOF was tested for adsorption and photocatalytic testing using the same methodology as that employed for CeO_2 materials.

Toxicity evaluation

According to OECD procedure No. 236, the Fish Embryo Toxicity (FET) test (Danio rerio) was used to evaluate the toxicity of the TC solutions that were the subject of this study (OECD, 2013). The test was performed in 24-well microplates, in three replicates. Twenty wells (i.e., TC solution before and after light exposure in the presence of a photocatalyst) were filled with 2 mL of the test solution at 30 mg L^{-1}, and four wells were filled with water (internal plate control). Immediately after the collection of the fertilized eggs, the tests began, in each well an egg was placed, which was kept in a climatized chamber (SL-24 Solab Científica, Brazil) at 25 °C for 144 h. For 168 hours, test solutions were applied to the zebrafish embryos, and any deformities were observed under a stereomicroscope daily. The University of Brasilia's ethical committee gave its approval to this study (reference number 100226/2014).

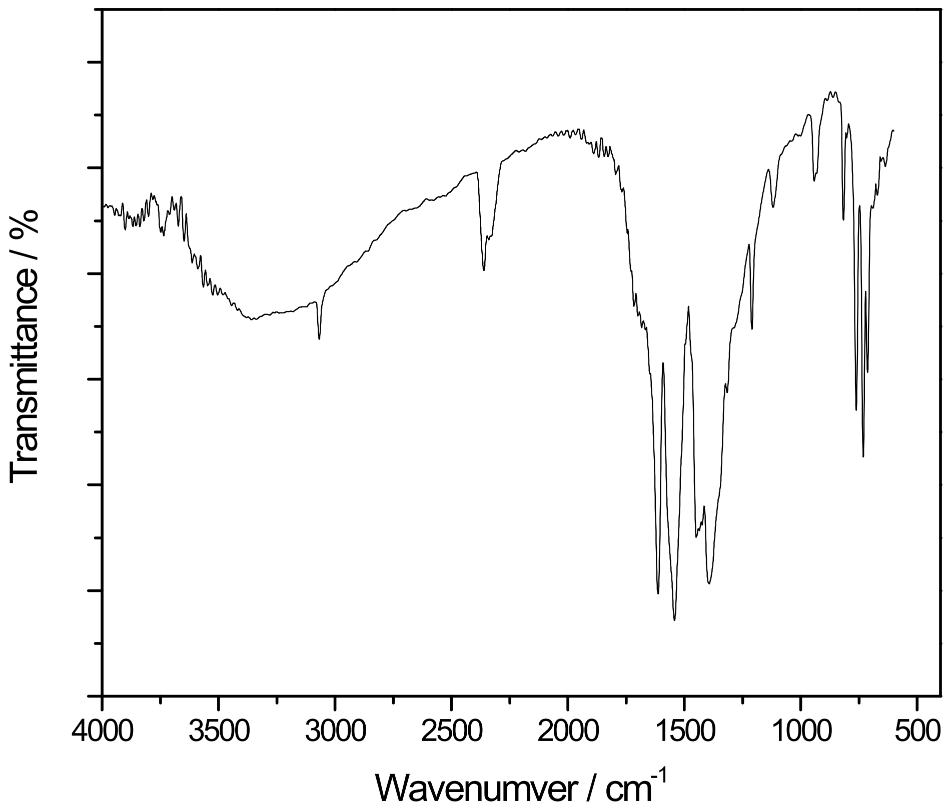

Figure S1. Vibrational infrared spectra (FTIR) for cerium metal-organic framework Ce-BTC.

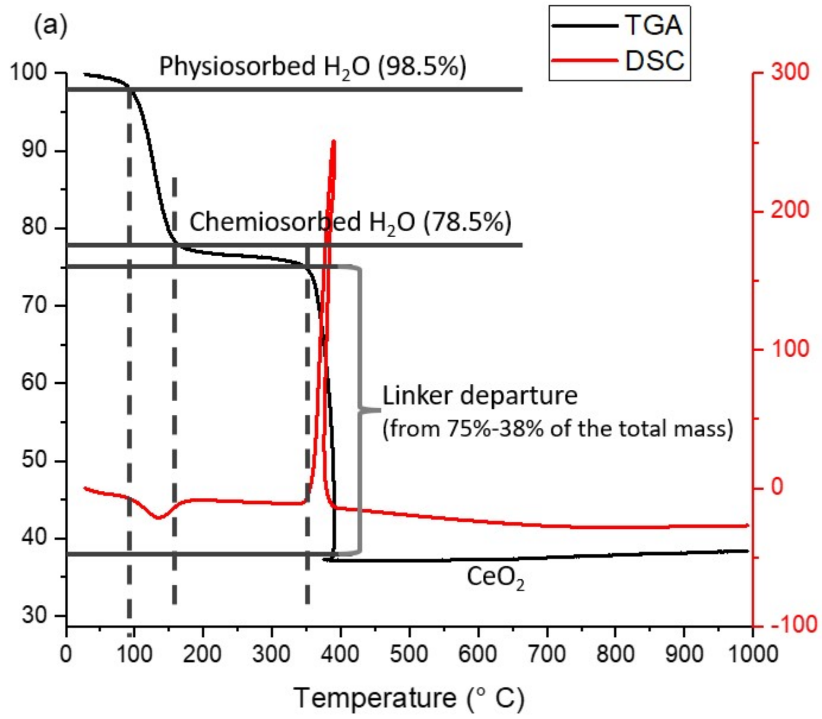

Figure S2: TGA/DSC analysis of Ce-MOF

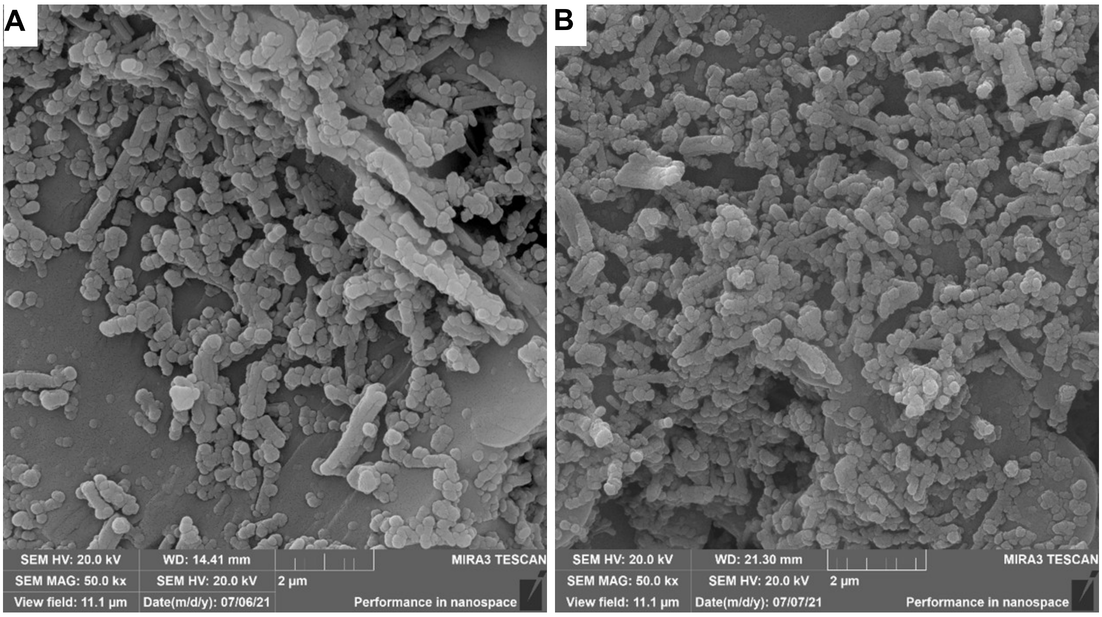

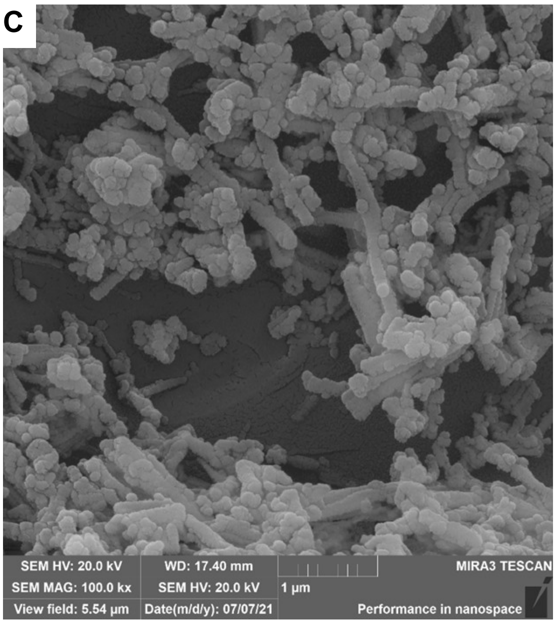

Fig S*3*: (A) Morphology of the oxides of oxides obtained by the Ce-MOF calcination at (a) 500, (b) 700 and (C) 900 °C.

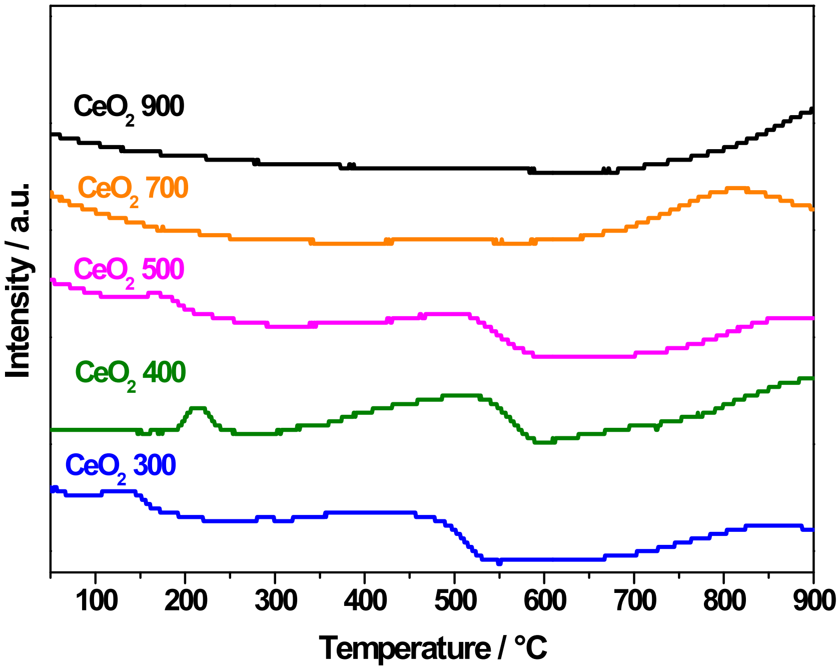

Fig S4: H_2 MS Signal for all the CeO_2 samples.

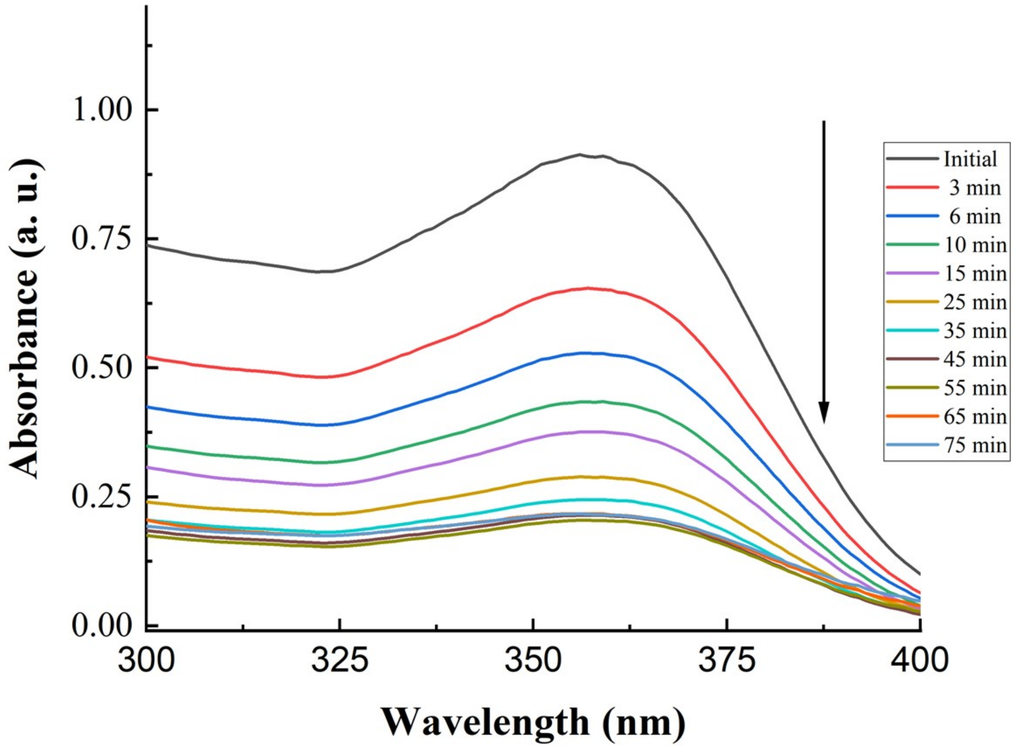

Fig S5: UV-Vis spectrum for Tetracycline (30 mg L^{-1}) as a function of adsorption time on CeO_2 (300 ºC), at room temperature.

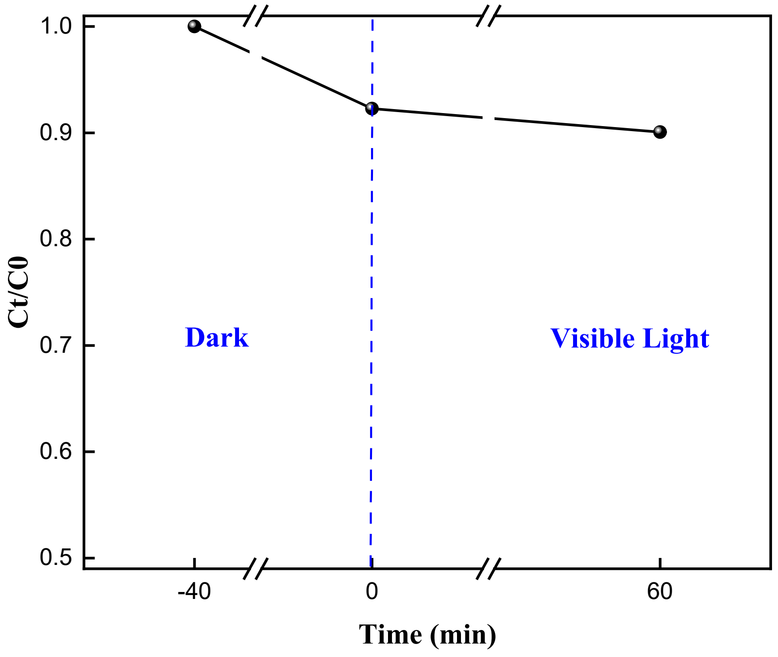

Fig S6: TC degradation performance of the photocatalyst Ce-MOF under simulative sunlight irradiation for 30 mg L^{-1} TCs.

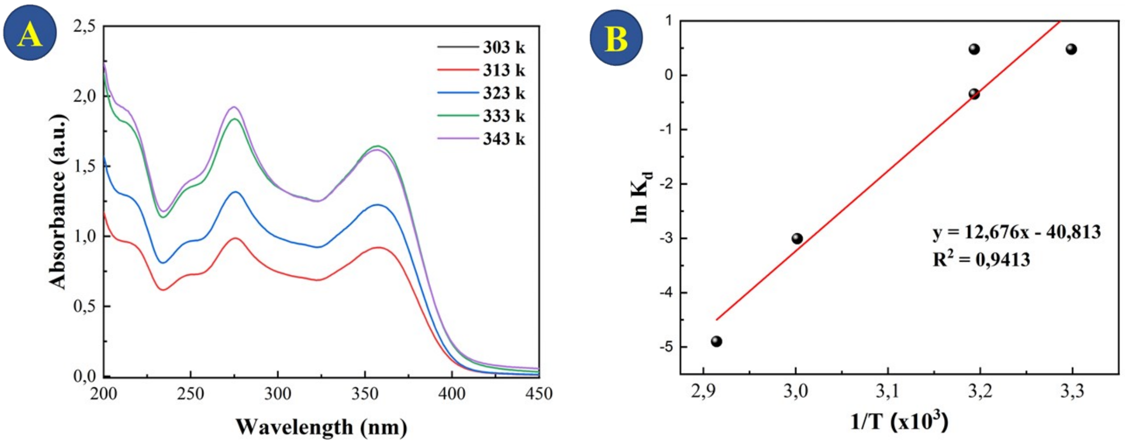

Fig S7: (A) TC adsorption (50 mg L^{-1}) at different temperatures; (B) Plot of *ln* Kd vs 1/T used to calculate the thermodynamic parameters of adsorption.

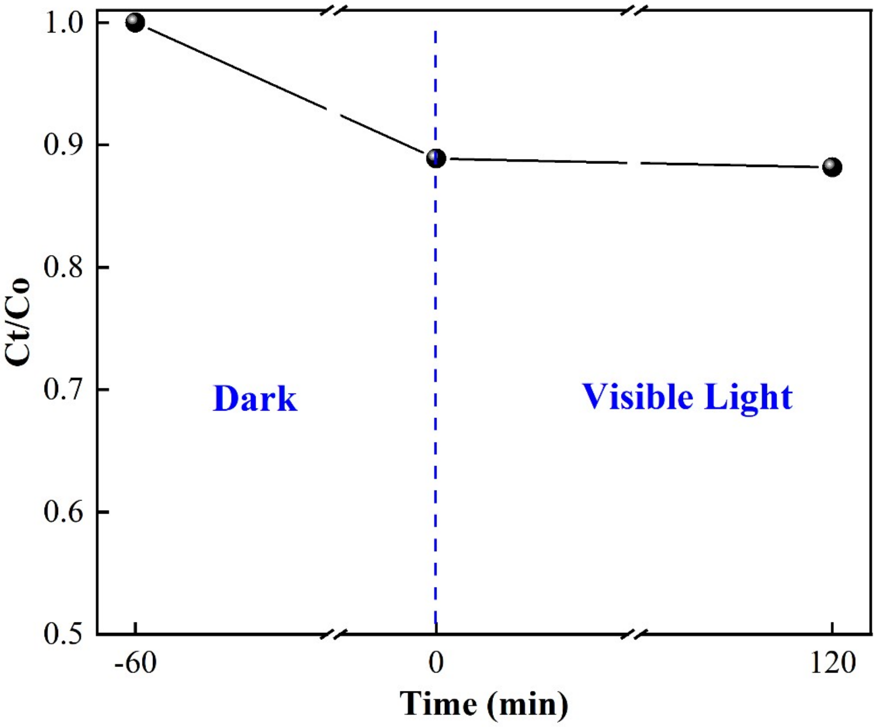

Fig S8: TC degradation performance of the photocatalyst (the commercial CeO_2) under simulative sunlight irradiation for 30 mg L^{-1} TCs.

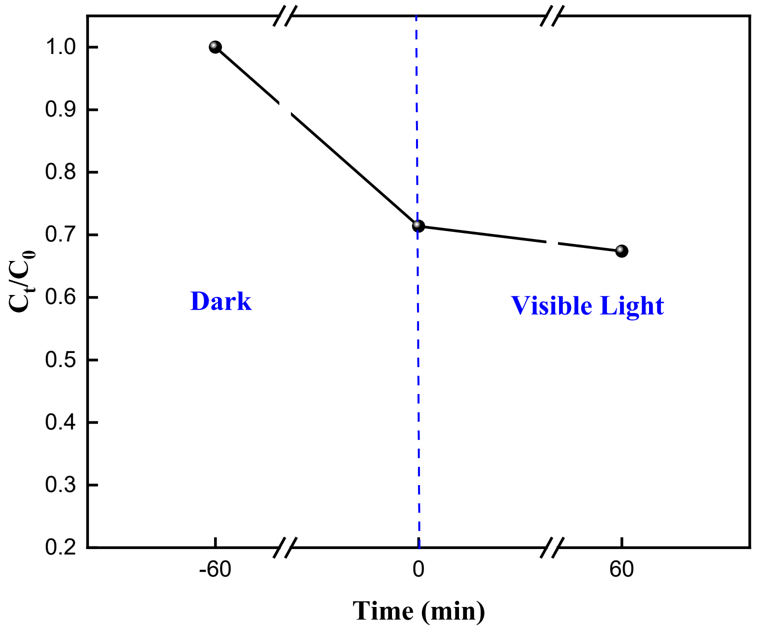

Fig S9: TC degradation performance in the reuse test for CeO_2-300 under simulative sunlight irradiation for 30 mg L^{-1} TCs. The composite CeO_2@TC was maintained in water by irradiation for 60 min before performing the reuse test.

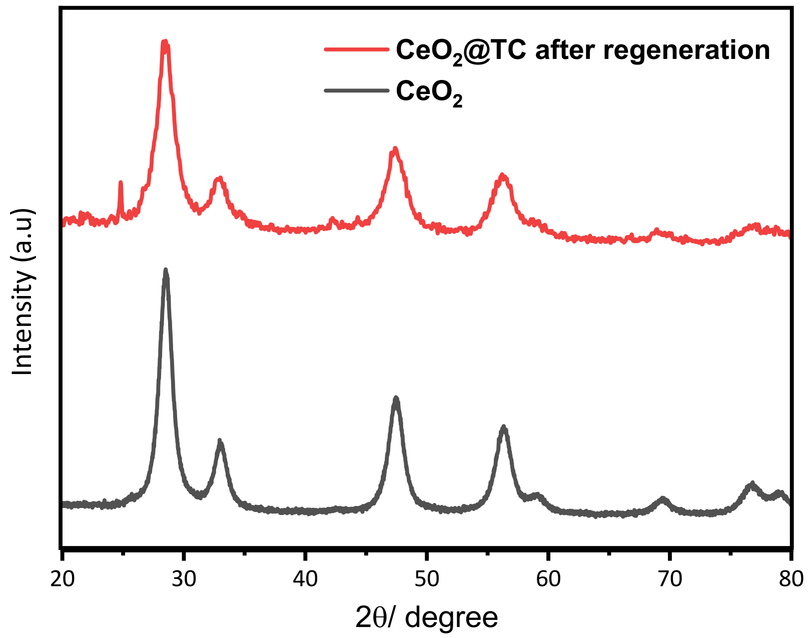

Fig S10: XRD pattern of the composite CeO_2@TC after regeneration for the reuse test.

a)

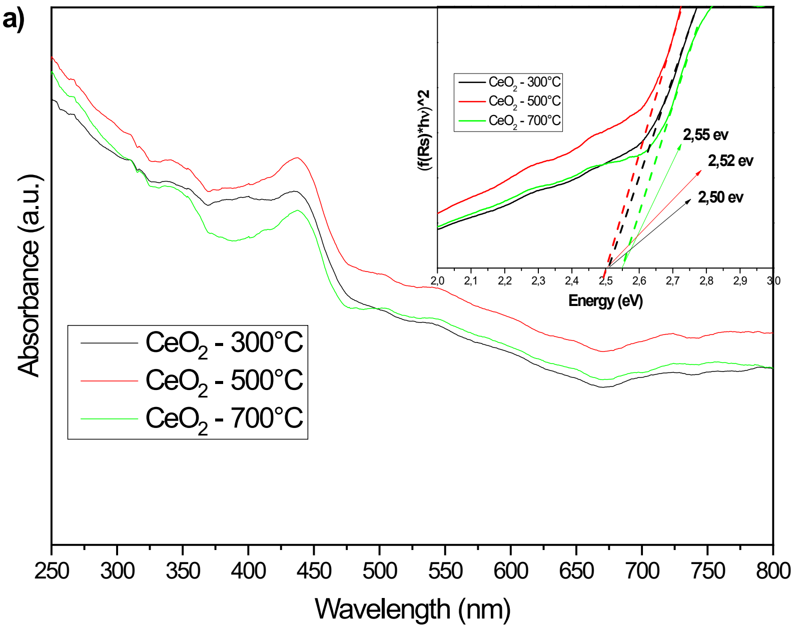

b)

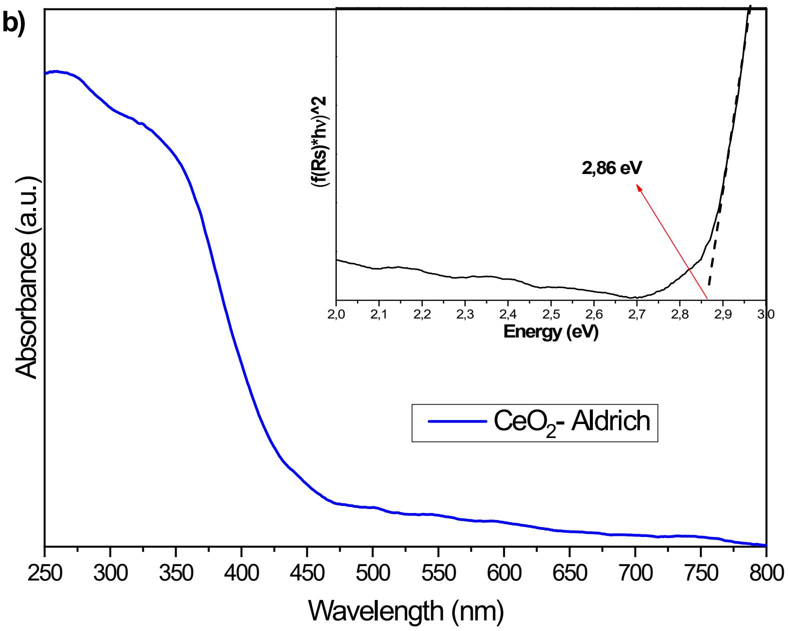

Fig S11: Transmittance spectrum and band-gap calculations for the (a) CeO_2-300, CeO_2-500, CeO_2-700 and (b) CeO_2 commercial.

Table S1: Peak positions and Miller indices for the powder XRD pattern of La(BTC)·$6H_2O$ (CCDC 290771).

$2\theta/°$	hkl
9.799	020
10.085	110
13.148	-111
16.985	021
17.182	130
17.676	200
18.967	-221
19.159	-131
19.67	040
20.248	220
20.405	111
23.665	-3-11
24.131	041
24.531	-202
24.76	131
25.182	-112
25.586	-2-41
26.231	150
26.473	-222
26.564	240
27.11	310
27.535	-3-31
27.594	-151
27.887	002
27.919	-312
28.863	-132
29.234	221
29.625	022
29.692	060
30.576	330
31.302	-332
31.638	-242
31.819	151
32.735	-402
32.795	-4-21
32.895	061
33.707	12
34.004	-2-61
34.018	241
34.068	-3-51
34.25	-4-22
34.359	042
34.766	260

Table S2: Comparison of adsorption and photocatalysis process for the oxides (CeO_2-300, CeO_2-500, CeO_2-700), for CeO_2 commercial, under TC solution of 30 mg.L^{-1}.

Samples	Adsorption (%)	Photocatalysis (%)	Total Removal (%)
CeO_2-300	60	38	98
CeO_2-500	40	25	65
CeO_2-700	15	20	35
CeO_2-commercial	10	0	10

Table S3: Potential zeta values for the oxides (CeO_2-300, CeO_2-500, CeO_2-700), for CeO_2 commercial, Ce-MOF and TC solution of 30 mg.L^{-1}.

Samples	Zeta Potential (mV)
CeO_2-300	+33.0
CeO_2-500	+26.7
CeO_2-700	-6.81
CeO_2-commercial	+5.40
Ce-MOF	-13.00
TC	-10.0
